# 4-Benzyl-4-methyl­morpholinium hexa­fluoro­phosphate

**DOI:** 10.1107/S1600536810038432

**Published:** 2010-09-30

**Authors:** Qiu-hong Su, Hong-Jun Zang, Tian-lin Xu, Yuan-Lin Ren

**Affiliations:** a Department of Enviromental and Chemistry Engineering, Tianjin Polytechnic University, State Key Laboratory of Hollow Fiber Membrane Materials and Processes, Tianjin 300160, People’s Republic of China; b Department of Textiles, Tianjin Polytechnic University, State Key Laboratory of Hollow Fiber Membrane Materials and Processes, Tianjin 300160, People’s Republic of China

## Abstract

In the title compound, C_12_H_18_NO^+^·PF_6_^−^, the asymmetric unit consists of two cation–anion pairs. The six F atoms of one anion are disordered over two sets of sites in a 0.592 (6):0.408 (6) ratio. The morpholinium rings adopt chair conformations.

## Related literature

Ionic liquids based on the morpholinium cation are favored becaused of their low cost, easy synthesis, and electrochemical stability, see: Kim *et al.* (2006 ▶).
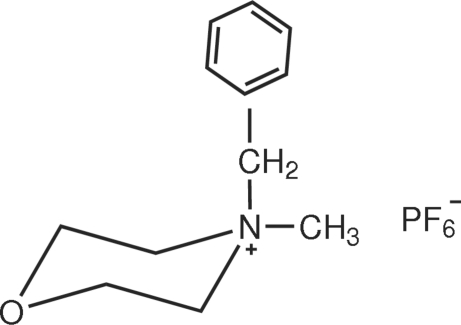


## Experimental

### 

#### Crystal data

C_12_H_18_NO^+^·PF_6_^−^
*M* *_r_* = 337.24
Triclinic, 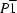

*a* = 9.7268 (14) Å
*b* = 10.7183 (16) Å
*c* = 14.537 (2) Å
α = 104.307 (5)°
β = 96.816 (8)°
γ = 95.633 (7)°
*V* = 1445.3 (4) Å^3^
*Z* = 4
Mo *K*α radiation
μ = 0.26 mm^−1^
*T* = 113 K
0.22 × 0.14 × 0.12 mm

#### Data collection

Rigaku Saturn diffractometer
Absorption correction: multi-scan (*CrystalClear*; Rigaku/MSC, 2005 ▶) *T*_min_ = 0.946, *T*_max_ = 0.970
17415 measured reflections
6387 independent reflections
4730 reflections with *I* > 2σ(*I*)
*R*_int_ = 0.043

#### Refinement

*R*[*F* ^2^ > 2σ(*F* ^2^)] = 0.049
*wR*(*F* ^2^) = 0.114
*S* = 1.03
6387 reflections
401 parameters
84 restraints
H-atom parameters constrained
Δρ_max_ = 0.53 e Å^−3^
Δρ_min_ = −0.40 e Å^−3^

### 

Data collection: *CrystalClear* (Rigaku/MSC, 2005 ▶); cell refinement: *CrystalClear*; data reduction: *CrystalClear*; program(s) used to solve structure: *SHELXS97* (Sheldrick, 2008 ▶); program(s) used to refine structure: *SHELXS97* (Sheldrick, 2008 ▶); molecular graphics: *SHELXTL* (Sheldrick, 2008 ▶); software used to prepare material for publication: *SHELXTL*.

## Supplementary Material

Crystal structure: contains datablocks I, global. DOI: 10.1107/S1600536810038432/jh2209sup1.cif

Structure factors: contains datablocks I. DOI: 10.1107/S1600536810038432/jh2209Isup2.hkl

Additional supplementary materials:  crystallographic information; 3D view; checkCIF report

## Figures and Tables

**Fig. 1.**
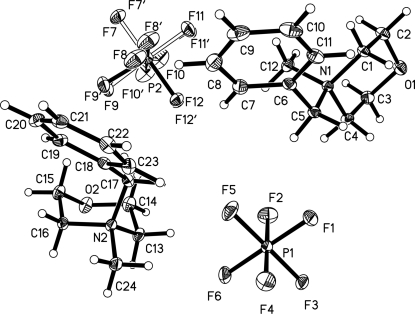
A view of the Structure of (I), Showing the atom-numbering scheme. Dispacement ellipsoids are drawn at the 30% probability level.

## supplementary crystallographic information

### Comment

Room-temperature ionic liquids (RTILs) consist of organic cation and anion which exist in liquid state at room temperature or below 100 °C. They are widely used in various fields of electrochemistry and chemistry because of their unique properties such as nonvolatility and nonflammability. In particular, ILs based on the morpholinium cation are favored becaused of their low cost, easy synthesis, and electrochemical stability (Kim *et al.*, 2006). So far, only a few crystallographic studies have been performed on salts. We report here a new example structure of this class.

The molecular structure of (I) is shown in Fig. 1. For the title compound two crystallographically independent molecules are present in the asymmetric unit of the cell. The morpholine unit adopts a chair conformation. Disorder model was introduced for the anion, in which the six fluorine atoms are all disordered over two positions. The bond distances and angles in the cation are normal within experimental error.

### Experimental

To a magnetically stirred solution of the 4-benzyl-4-methylmorpholinium chloride (2.29 g, 10 mmol) in acetonitrile (20 ml) was added potassium hexafluorophosphate (1.86 g, 10 mmol). The mixture was stirred at room temperrature for 72 h, and the KCl filtered from the reaction mixture. The solvent was removed under reduced pressure. The residue was washed by ether and then recrystallized from hot ethanol to afford the product. A single-crystal was obtained by slow evaporation of a EtOH solution.

### Refinement

The H atoms bonded to C atoms were included in the refinement in the riding model approximation, with C–H = 0.93–0.97 Å and *U*_iso_ (H) = 1.2 *U*_eq_ (C atom). For the H atoms attached to C atoms of methyl groups, their *U*_iso_(H) =1.5*U*_eq_(C).

### Crystal data

**Table:**

C_12_H_18_NO^+^·PF_6_^−^	*Z* = 4
*M**_r_* = 337.24	*F*(000) = 696
Triclinic, *P*1	*D*_x_ = 1.550 Mg m^−^^3^
*a* = 9.7268 (14) Å	Mo *K*α radiation, λ = 0.71070 Å
*b* = 10.7183 (16) Å	Cell parameters from 4804 reflections
*c* = 14.537 (2) Å	θ = 1.5–27.2°
α = 104.307 (5)°	µ = 0.26 mm^−^^1^
β = 96.816 (8)°	*T* = 113 K
γ = 95.633 (7)°	Block, colorless
*V* = 1445.3 (4) Å^3^	0.22 × 0.14 × 0.12 mm

### Data collection

**Table:**

Rigaku Saturn diffractometer	6387 independent reflections
Radiation source: rotating anode	4730 reflections with *I* > 2σ(*I*)
confocal	*R*_int_ = 0.043
Detector resolution: 14.63 pixels mm^-1^	θ_max_ = 27.2°, θ_min_ = 1.5°
ω scans	*h* = −12→12
Absorption correction: multi-scan (*CrystalClear*; Rigaku/MSC, 2005)	*k* = −13→13
*T*_min_ = 0.946, *T*_max_ = 0.970	*l* = −18→18
17415 measured reflections	

### Refinement

**Table:**

Refinement on *F*^2^	Secondary atom site location: difference Fourier map
Least-squares matrix: full	Hydrogen site location: inferred from neighbouring sites
*R*[*F*^2^ > 2σ(*F*^2^)] = 0.049	H-atom parameters constrained
*wR*(*F*^2^) = 0.114	*w* = 1/[σ^2^(*F*_o_^2^) + (0.0518*P*)^2^] where *P* = (*F*_o_^2^ + 2*F*_c_^2^)/3
*S* = 1.03	(Δ/σ)_max_ = 0.002
6387 reflections	Δρ_max_ = 0.53 e Å^−^^3^
401 parameters	Δρ_min_ = −0.40 e Å^−^^3^
84 restraints	Extinction correction: *SHELXS97* (Sheldrick, 2008), Fc^*^=kFc[1+0.001xFc^2^λ^3^/sin(2θ)]^-1/4^
Primary atom site location: structure-invariant direct methods	Extinction coefficient: 0.0123 (11)

### Special details

**Table:**

Geometry. All e.s.d.'s (except the e.s.d. in the dihedral angle between two l.s. planes) are estimated using the full covariance matrix. The cell e.s.d.'s are taken into account individually in the estimation of e.s.d.'s in distances, angles and torsion angles; correlations between e.s.d.'s in cell parameters are only used when they are defined by crystal symmetry. An approximate (isotropic) treatment of cell e.s.d.'s is used for estimating e.s.d.'s involving l.s. planes.
Refinement. Refinement of *F*^2^ against ALL reflections. The weighted *R*-factor *wR* and goodness of fit *S* are based on *F*^2^, conventional *R*-factors *R* are based on *F*, with *F* set to zero for negative *F*^2^. The threshold expression of *F*^2^ > σ(*F*^2^) is used only for calculating *R*-factors(gt) *etc*. and is not relevant to the choice of reflections for refinement. *R*-factors based on *F*^2^ are statistically about twice as large as those based on *F*, and *R*- factors based on ALL data will be even larger.

### Fractional atomic coordinates and isotropic or equivalent isotropic displacement parameters (Å^2^)

**Table:**

	*x*	*y*	*z*	*U*_iso_*/*U*_eq_	Occ. (<1)
P1	0.30855 (6)	0.37650 (5)	0.09741 (4)	0.02340 (16)	
P2	0.78163 (6)	0.24754 (6)	0.46477 (4)	0.02855 (17)	
F1	0.42358 (14)	0.39964 (13)	0.03267 (9)	0.0368 (4)	
F2	0.40636 (15)	0.46701 (15)	0.19023 (9)	0.0463 (4)	
F3	0.23988 (14)	0.49934 (12)	0.07859 (9)	0.0342 (3)	
F4	0.20934 (16)	0.28651 (14)	0.00513 (10)	0.0477 (4)	
F5	0.37474 (17)	0.25367 (14)	0.11626 (10)	0.0485 (4)	
F6	0.19177 (15)	0.35492 (14)	0.16311 (10)	0.0449 (4)	
F7'	0.8866 (8)	0.1865 (7)	0.5320 (5)	0.0485 (9)	0.592 (6)
F8'	0.8069 (6)	0.1427 (5)	0.3741 (3)	0.0583 (12)	0.592 (6)
F9'	0.6552 (6)	0.1530 (5)	0.4805 (4)	0.0444 (11)	0.592 (6)
F10'	0.7621 (6)	0.3542 (5)	0.5615 (4)	0.0595 (14)	0.592 (6)
F11'	0.9139 (4)	0.3438 (5)	0.4548 (4)	0.0648 (13)	0.592 (6)
F12'	0.6806 (7)	0.3126 (7)	0.4031 (6)	0.0322 (4)	0.592 (6)
F7	0.8809 (11)	0.1709 (11)	0.5169 (8)	0.0485 (9)	0.408 (6)
F8	0.7411 (7)	0.1201 (7)	0.3742 (5)	0.0583 (12)	0.408 (6)
F9	0.6530 (9)	0.1932 (7)	0.5062 (6)	0.0444 (11)	0.408 (6)
F10	0.8134 (9)	0.3697 (7)	0.5478 (6)	0.0595 (14)	0.408 (6)
F11	0.9009 (7)	0.2907 (8)	0.4109 (5)	0.0648 (13)	0.408 (6)
F12	0.6762 (11)	0.3186 (11)	0.4054 (8)	0.0322 (4)	0.408 (6)
O1	1.00560 (16)	0.81093 (14)	0.22635 (11)	0.0328 (4)	
O2	0.27675 (18)	0.27512 (15)	0.54849 (10)	0.0333 (4)	
N1	0.85004 (17)	0.55784 (16)	0.20963 (12)	0.0205 (4)	
N2	0.23494 (18)	0.16209 (16)	0.34181 (12)	0.0215 (4)	
C1	0.9767 (2)	0.5774 (2)	0.16140 (15)	0.0235 (5)	
H1A	0.9459	0.5845	0.0957	0.028*	
H1B	1.0272	0.5007	0.1559	0.028*	
C2	1.0748 (2)	0.6979 (2)	0.21631 (17)	0.0295 (5)	
H2A	1.1104	0.6888	0.2807	0.035*	
H2B	1.1556	0.7076	0.1820	0.035*	
C3	0.8918 (2)	0.8002 (2)	0.27847 (16)	0.0304 (5)	
H3A	0.8457	0.8797	0.2861	0.037*	
H3B	0.9276	0.7928	0.3433	0.037*	
C4	0.7860 (2)	0.6831 (2)	0.22778 (16)	0.0262 (5)	
H4A	0.7435	0.6950	0.1657	0.031*	
H4B	0.7107	0.6769	0.2673	0.031*	
C5	0.7400 (2)	0.4531 (2)	0.14237 (15)	0.0234 (5)	
H5A	0.6536	0.4513	0.1721	0.028*	
H5B	0.7180	0.4783	0.0817	0.028*	
C6	0.7801 (2)	0.3181 (2)	0.11851 (15)	0.0237 (5)	
C7	0.7297 (2)	0.2279 (2)	0.16439 (17)	0.0317 (5)	
H7	0.6761	0.2540	0.2147	0.038*	
C8	0.7564 (3)	0.1000 (2)	0.13772 (19)	0.0406 (6)	
H8	0.7215	0.0391	0.1697	0.049*	
C9	0.8342 (3)	0.0617 (2)	0.0645 (2)	0.0433 (7)	
H9	0.8526	−0.0257	0.0457	0.052*	
C10	0.8852 (3)	0.1513 (3)	0.01851 (19)	0.0415 (7)	
H10	0.9394	0.1252	−0.0314	0.050*	
C11	0.8581 (2)	0.2777 (2)	0.04457 (16)	0.0314 (6)	
H11	0.8927	0.3380	0.0120	0.038*	
C12	0.8884 (2)	0.5197 (2)	0.30086 (14)	0.0262 (5)	
H12A	0.9362	0.4423	0.2874	0.039*	
H12B	0.8035	0.5008	0.3279	0.039*	
H12C	0.9505	0.5912	0.3470	0.039*	
C13	0.2326 (2)	0.3049 (2)	0.38787 (16)	0.0275 (5)	
H13A	0.1351	0.3204	0.3943	0.033*	
H13B	0.2675	0.3568	0.3457	0.033*	
C14	0.3215 (3)	0.3497 (2)	0.48603 (16)	0.0321 (6)	
H14A	0.4204	0.3410	0.4792	0.038*	
H14B	0.3151	0.4426	0.5144	0.038*	
C15	0.2877 (3)	0.1415 (2)	0.50940 (15)	0.0297 (5)	
H15A	0.2595	0.0912	0.5543	0.036*	
H15B	0.3861	0.1314	0.5018	0.036*	
C16	0.1962 (2)	0.0883 (2)	0.41301 (15)	0.0245 (5)	
H16A	0.2060	−0.0045	0.3877	0.029*	
H16B	0.0972	0.0943	0.4212	0.029*	
C17	0.3792 (2)	0.1439 (2)	0.31398 (15)	0.0233 (5)	
H17A	0.4005	0.2021	0.2728	0.028*	
H17B	0.4486	0.1724	0.3731	0.028*	
C18	0.3991 (2)	0.0077 (2)	0.26190 (15)	0.0222 (5)	
C19	0.4247 (2)	−0.0859 (2)	0.31083 (15)	0.0259 (5)	
H19	0.4245	−0.0661	0.3782	0.031*	
C20	0.4506 (2)	−0.2080 (2)	0.26217 (16)	0.0305 (5)	
H20	0.4643	−0.2723	0.2959	0.037*	
C21	0.4564 (2)	−0.2362 (2)	0.16470 (16)	0.0281 (5)	
H21	0.4756	−0.3193	0.1316	0.034*	
C22	0.4341 (2)	−0.1434 (2)	0.11574 (16)	0.0294 (5)	
H22	0.4394	−0.1622	0.0490	0.035*	
C23	0.4040 (2)	−0.0223 (2)	0.16372 (15)	0.0278 (5)	
H23	0.3866	0.0405	0.1292	0.033*	
C24	0.1262 (2)	0.1182 (2)	0.25385 (15)	0.0289 (5)	
H24A	0.1221	0.0244	0.2269	0.043*	
H24B	0.1508	0.1637	0.2061	0.043*	
H24C	0.0350	0.1380	0.2712	0.043*	

### Atomic displacement parameters (Å^2^)

**Table:**

	*U*^11^	*U*^22^	*U*^33^	*U*^12^	*U*^13^	*U*^23^
P1	0.0243 (3)	0.0239 (3)	0.0254 (3)	0.0053 (2)	0.0057 (3)	0.0112 (2)
P2	0.0243 (3)	0.0329 (4)	0.0321 (4)	0.0022 (3)	0.0026 (3)	0.0165 (3)
F1	0.0325 (8)	0.0462 (9)	0.0401 (8)	0.0109 (7)	0.0181 (7)	0.0186 (7)
F2	0.0434 (9)	0.0533 (10)	0.0331 (8)	0.0054 (7)	−0.0054 (7)	0.0000 (7)
F3	0.0361 (8)	0.0347 (8)	0.0433 (8)	0.0166 (6)	0.0148 (7)	0.0229 (6)
F4	0.0494 (10)	0.0389 (9)	0.0444 (9)	−0.0092 (7)	−0.0088 (7)	0.0046 (7)
F5	0.0645 (11)	0.0424 (9)	0.0555 (10)	0.0321 (8)	0.0210 (8)	0.0288 (8)
F6	0.0417 (9)	0.0553 (10)	0.0563 (10)	0.0147 (7)	0.0240 (8)	0.0378 (8)
F7'	0.0379 (11)	0.0567 (19)	0.058 (2)	0.0078 (12)	−0.0078 (12)	0.0350 (18)
F8'	0.076 (3)	0.070 (2)	0.0396 (10)	0.046 (2)	0.020 (2)	0.0151 (12)
F9'	0.0339 (9)	0.047 (3)	0.057 (3)	−0.0077 (18)	0.0016 (16)	0.031 (2)
F10'	0.084 (4)	0.0476 (16)	0.0360 (16)	0.016 (2)	−0.0128 (19)	−0.0014 (13)
F11'	0.0255 (12)	0.083 (3)	0.101 (4)	−0.0102 (18)	0.000 (2)	0.065 (3)
F12'	0.0292 (8)	0.0341 (10)	0.0355 (8)	0.0051 (6)	−0.0023 (7)	0.0162 (7)
F7	0.0379 (11)	0.0567 (19)	0.058 (2)	0.0078 (12)	−0.0078 (12)	0.0350 (18)
F8	0.076 (3)	0.070 (2)	0.0396 (10)	0.046 (2)	0.020 (2)	0.0151 (12)
F9	0.0339 (9)	0.047 (3)	0.057 (3)	−0.0077 (18)	0.0016 (16)	0.031 (2)
F10	0.084 (4)	0.0476 (16)	0.0360 (16)	0.016 (2)	−0.0128 (19)	−0.0014 (13)
F11	0.0255 (12)	0.083 (3)	0.101 (4)	−0.0102 (18)	0.000 (2)	0.065 (3)
F12	0.0292 (8)	0.0341 (10)	0.0355 (8)	0.0051 (6)	−0.0023 (7)	0.0162 (7)
O1	0.0361 (10)	0.0228 (9)	0.0409 (10)	0.0034 (7)	0.0130 (8)	0.0079 (7)
O2	0.0499 (11)	0.0283 (9)	0.0239 (8)	0.0131 (8)	0.0112 (8)	0.0051 (7)
N1	0.0187 (9)	0.0237 (10)	0.0204 (9)	0.0049 (7)	0.0041 (8)	0.0067 (7)
N2	0.0240 (10)	0.0193 (9)	0.0218 (9)	0.0034 (7)	0.0019 (8)	0.0069 (7)
C1	0.0192 (11)	0.0264 (12)	0.0259 (12)	0.0048 (9)	0.0083 (9)	0.0057 (9)
C2	0.0245 (13)	0.0277 (12)	0.0353 (13)	0.0032 (10)	0.0090 (10)	0.0041 (10)
C3	0.0327 (14)	0.0276 (12)	0.0336 (13)	0.0102 (10)	0.0116 (11)	0.0073 (10)
C4	0.0249 (12)	0.0272 (12)	0.0296 (12)	0.0107 (10)	0.0064 (10)	0.0092 (10)
C5	0.0174 (11)	0.0311 (12)	0.0212 (11)	0.0019 (9)	0.0002 (9)	0.0076 (9)
C6	0.0173 (11)	0.0285 (12)	0.0222 (11)	−0.0007 (9)	−0.0019 (9)	0.0046 (9)
C7	0.0268 (13)	0.0339 (13)	0.0311 (13)	−0.0031 (10)	−0.0029 (10)	0.0086 (10)
C8	0.0378 (15)	0.0310 (14)	0.0484 (16)	−0.0052 (11)	−0.0098 (13)	0.0133 (12)
C9	0.0311 (15)	0.0283 (14)	0.0589 (18)	0.0046 (11)	−0.0114 (13)	−0.0017 (13)
C10	0.0254 (14)	0.0392 (15)	0.0483 (16)	0.0031 (11)	0.0012 (12)	−0.0075 (13)
C11	0.0241 (13)	0.0338 (13)	0.0313 (13)	−0.0005 (10)	0.0035 (10)	0.0013 (10)
C12	0.0279 (13)	0.0285 (12)	0.0220 (11)	0.0041 (10)	−0.0006 (10)	0.0080 (9)
C13	0.0340 (14)	0.0200 (11)	0.0316 (12)	0.0092 (10)	0.0069 (11)	0.0091 (9)
C14	0.0435 (15)	0.0215 (12)	0.0299 (13)	0.0066 (11)	0.0067 (11)	0.0027 (10)
C15	0.0414 (15)	0.0279 (13)	0.0245 (12)	0.0118 (11)	0.0099 (11)	0.0102 (10)
C16	0.0255 (12)	0.0256 (12)	0.0273 (12)	0.0053 (9)	0.0097 (10)	0.0123 (9)
C17	0.0225 (12)	0.0244 (11)	0.0218 (11)	−0.0016 (9)	0.0035 (9)	0.0056 (9)
C18	0.0188 (11)	0.0231 (11)	0.0223 (11)	−0.0005 (9)	0.0023 (9)	0.0035 (9)
C19	0.0226 (12)	0.0334 (13)	0.0227 (11)	0.0084 (10)	0.0024 (9)	0.0078 (10)
C20	0.0273 (13)	0.0316 (13)	0.0363 (13)	0.0118 (10)	0.0052 (11)	0.0129 (11)
C21	0.0214 (12)	0.0256 (12)	0.0343 (13)	0.0048 (9)	0.0051 (10)	0.0014 (10)
C22	0.0297 (13)	0.0318 (13)	0.0230 (12)	−0.0008 (10)	0.0051 (10)	0.0014 (10)
C23	0.0320 (13)	0.0264 (12)	0.0241 (12)	0.0014 (10)	0.0031 (10)	0.0063 (10)
C24	0.0279 (13)	0.0281 (12)	0.0287 (12)	−0.0015 (10)	−0.0056 (10)	0.0102 (10)

### Geometric parameters (Å, °)

**Table:**

P1—F1	1.5843 (13)	C5—H5B	0.9900
P1—F5	1.5893 (13)	C6—C7	1.386 (3)
P1—F4	1.5910 (14)	C6—C11	1.395 (3)
P1—F2	1.5919 (14)	C7—C8	1.389 (3)
P1—F3	1.6005 (13)	C7—H7	0.9500
P1—F6	1.6035 (14)	C8—C9	1.382 (4)
P2—F10	1.523 (6)	C8—H8	0.9500
P2—F8'	1.568 (4)	C9—C10	1.384 (4)
P2—F11	1.569 (5)	C9—H9	0.9500
P2—F7	1.572 (6)	C10—C11	1.374 (3)
P2—F9	1.573 (6)	C10—H10	0.9500
P2—F12'	1.578 (5)	C11—H11	0.9500
P2—F9'	1.590 (4)	C12—H12A	0.9800
P2—F11'	1.612 (4)	C12—H12B	0.9800
P2—F10'	1.623 (4)	C12—H12C	0.9800
P2—F7'	1.624 (4)	C13—C14	1.517 (3)
P2—F8	1.624 (6)	C13—H13A	0.9900
P2—F12	1.626 (6)	C13—H13B	0.9900
O1—C3	1.425 (3)	C14—H14A	0.9900
O1—C2	1.427 (3)	C14—H14B	0.9900
O2—C15	1.424 (2)	C15—C16	1.513 (3)
O2—C14	1.427 (3)	C15—H15A	0.9900
N1—C12	1.498 (3)	C15—H15B	0.9900
N1—C1	1.510 (2)	C16—H16A	0.9900
N1—C4	1.513 (3)	C16—H16B	0.9900
N1—C5	1.528 (3)	C17—C18	1.512 (3)
N2—C24	1.503 (3)	C17—H17A	0.9900
N2—C16	1.509 (3)	C17—H17B	0.9900
N2—C13	1.515 (3)	C18—C19	1.391 (3)
N2—C17	1.524 (3)	C18—C23	1.391 (3)
C1—C2	1.512 (3)	C19—C20	1.387 (3)
C1—H1A	0.9900	C19—H19	0.9500
C1—H1B	0.9900	C20—C21	1.383 (3)
C2—H2A	0.9900	C20—H20	0.9500
C2—H2B	0.9900	C21—C22	1.377 (3)
C3—C4	1.512 (3)	C21—H21	0.9500
C3—H3A	0.9900	C22—C23	1.391 (3)
C3—H3B	0.9900	C22—H22	0.9500
C4—H4A	0.9900	C23—H23	0.9500
C4—H4B	0.9900	C24—H24A	0.9800
C5—C6	1.503 (3)	C24—H24B	0.9800
C5—H5A	0.9900	C24—H24C	0.9800
			
F1—P1—F5	90.51 (8)	O1—C2—H2A	109.4
F1—P1—F4	89.97 (8)	C1—C2—H2A	109.4
F5—P1—F4	90.45 (9)	O1—C2—H2B	109.4
F1—P1—F2	90.56 (8)	C1—C2—H2B	109.4
F5—P1—F2	89.86 (9)	H2A—C2—H2B	108.0
F4—P1—F2	179.38 (8)	O1—C3—C4	111.43 (17)
F1—P1—F3	90.16 (7)	O1—C3—H3A	109.3
F5—P1—F3	179.23 (8)	C4—C3—H3A	109.3
F4—P1—F3	89.18 (8)	O1—C3—H3B	109.3
F2—P1—F3	90.50 (8)	C4—C3—H3B	109.3
F1—P1—F6	179.29 (8)	H3A—C3—H3B	108.0
F5—P1—F6	90.15 (8)	C3—C4—N1	112.51 (18)
F4—P1—F6	90.26 (8)	C3—C4—H4A	109.1
F2—P1—F6	89.20 (8)	N1—C4—H4A	109.1
F3—P1—F6	89.18 (7)	C3—C4—H4B	109.1
F10—P2—F8'	157.7 (3)	N1—C4—H4B	109.1
F10—P2—F11	94.0 (3)	H4A—C4—H4B	107.8
F8'—P2—F11	64.7 (3)	C6—C5—N1	115.64 (17)
F10—P2—F7	93.1 (4)	C6—C5—H5A	108.4
F8'—P2—F7	81.6 (5)	N1—C5—H5A	108.4
F11—P2—F7	91.5 (4)	C6—C5—H5B	108.4
F10—P2—F9	92.6 (3)	N1—C5—H5B	108.4
F8'—P2—F9	109.1 (3)	H5A—C5—H5B	107.4
F11—P2—F9	172.8 (4)	C7—C6—C11	118.6 (2)
F7—P2—F9	91.0 (4)	C7—C6—C5	120.2 (2)
F10—P2—F12'	92.7 (6)	C11—C6—C5	120.9 (2)
F8'—P2—F12'	92.7 (3)	C6—C7—C8	120.9 (2)
F11—P2—F12'	87.2 (5)	C6—C7—H7	119.6
F7—P2—F12'	174.2 (6)	C8—C7—H7	119.6
F9—P2—F12'	89.6 (6)	C9—C8—C7	119.7 (3)
F10—P2—F9'	110.4 (3)	C9—C8—H8	120.1
F8'—P2—F9'	91.0 (2)	C7—C8—H8	120.1
F11—P2—F9'	155.6 (3)	C8—C9—C10	119.7 (2)
F7—P2—F9'	86.9 (6)	C8—C9—H9	120.1
F9—P2—F9'	18.2 (3)	C10—C9—H9	120.1
F12'—P2—F9'	91.9 (3)	C11—C10—C9	120.5 (2)
F10—P2—F11'	67.6 (3)	C11—C10—H10	119.7
F8'—P2—F11'	90.77 (19)	C9—C10—H10	119.7
F11—P2—F11'	26.5 (3)	C10—C11—C6	120.5 (2)
F7—P2—F11'	90.7 (5)	C10—C11—H11	119.7
F9—P2—F11'	160.1 (3)	C6—C11—H11	119.7
F12'—P2—F11'	90.7 (3)	N1—C12—H12A	109.5
F9'—P2—F11'	176.8 (3)	N1—C12—H12B	109.5
F10—P2—F10'	21.1 (4)	H12A—C12—H12B	109.5
F8'—P2—F10'	177.2 (3)	N1—C12—H12C	109.5
F11—P2—F10'	114.7 (3)	H12A—C12—H12C	109.5
F7—P2—F10'	95.7 (6)	H12B—C12—H12C	109.5
F9—P2—F10'	71.7 (3)	N2—C13—C14	111.92 (17)
F12'—P2—F10'	90.0 (3)	N2—C13—H13A	109.2
F9'—P2—F10'	89.7 (2)	C14—C13—H13A	109.2
F11'—P2—F10'	88.5 (2)	N2—C13—H13B	109.2
F10—P2—F7'	84.8 (6)	C14—C13—H13B	109.2
F8'—P2—F7'	89.7 (3)	H13A—C13—H13B	107.9
F11—P2—F7'	93.0 (5)	O2—C14—C13	111.06 (19)
F7—P2—F7'	8.3 (7)	O2—C14—H14A	109.4
F9—P2—F7'	90.4 (6)	C13—C14—H14A	109.4
F12'—P2—F7'	177.5 (4)	O2—C14—H14B	109.4
F9'—P2—F7'	88.9 (3)	C13—C14—H14B	109.4
F11'—P2—F7'	88.4 (3)	H14A—C14—H14B	108.0
F10'—P2—F7'	87.6 (3)	O2—C15—C16	111.24 (17)
F10—P2—F8	177.2 (4)	O2—C15—H15A	109.4
F8'—P2—F8	23.8 (2)	C16—C15—H15A	109.4
F11—P2—F8	86.7 (3)	O2—C15—H15B	109.4
F7—P2—F8	89.6 (4)	C16—C15—H15B	109.4
F9—P2—F8	86.6 (3)	H15A—C15—H15B	108.0
F12'—P2—F8	84.6 (5)	N2—C16—C15	111.46 (18)
F9'—P2—F8	68.9 (3)	N2—C16—H16A	109.3
F11'—P2—F8	113.2 (2)	C15—C16—H16A	109.3
F10'—P2—F8	157.7 (2)	N2—C16—H16B	109.3
F7'—P2—F8	97.9 (5)	C15—C16—H16B	109.3
F10—P2—F12	90.5 (4)	H16A—C16—H16B	108.0
F8'—P2—F12	95.1 (5)	C18—C17—N2	116.30 (17)
F11—P2—F12	88.5 (4)	C18—C17—H17A	108.2
F7—P2—F12	176.4 (5)	N2—C17—H17A	108.2
F9—P2—F12	88.6 (4)	C18—C17—H17B	108.2
F12'—P2—F12	2.4 (7)	N2—C17—H17B	108.2
F9'—P2—F12	91.7 (6)	H17A—C17—H17B	107.4
F11'—P2—F12	90.9 (5)	C19—C18—C23	118.67 (19)
F10'—P2—F12	87.6 (6)	C19—C18—C17	121.67 (19)
F7'—P2—F12	175.2 (6)	C23—C18—C17	119.42 (19)
F8—P2—F12	86.8 (4)	C20—C19—C18	120.6 (2)
C3—O1—C2	110.05 (17)	C20—C19—H19	119.7
C15—O2—C14	110.09 (15)	C18—C19—H19	119.7
C12—N1—C1	111.17 (16)	C21—C20—C19	120.1 (2)
C12—N1—C4	111.53 (16)	C21—C20—H20	119.9
C1—N1—C4	107.61 (16)	C19—C20—H20	119.9
C12—N1—C5	109.33 (16)	C22—C21—C20	119.8 (2)
C1—N1—C5	110.28 (15)	C22—C21—H21	120.1
C4—N1—C5	106.81 (16)	C20—C21—H21	120.1
C24—N2—C16	108.23 (17)	C21—C22—C23	120.2 (2)
C24—N2—C13	109.28 (16)	C21—C22—H22	119.9
C16—N2—C13	107.35 (15)	C23—C22—H22	119.9
C24—N2—C17	109.83 (16)	C22—C23—C18	120.5 (2)
C16—N2—C17	113.36 (16)	C22—C23—H23	119.8
C13—N2—C17	108.70 (16)	C18—C23—H23	119.8
N1—C1—C2	112.04 (16)	N2—C24—H24A	109.5
N1—C1—H1A	109.2	N2—C24—H24B	109.5
C2—C1—H1A	109.2	H24A—C24—H24B	109.5
N1—C1—H1B	109.2	N2—C24—H24C	109.5
C2—C1—H1B	109.2	H24A—C24—H24C	109.5
H1A—C1—H1B	107.9	H24B—C24—H24C	109.5
O1—C2—C1	110.99 (18)		
			
C12—N1—C1—C2	−70.4 (2)	C24—N2—C13—C14	170.01 (18)
C4—N1—C1—C2	52.0 (2)	C16—N2—C13—C14	52.8 (2)
C5—N1—C1—C2	168.14 (17)	C17—N2—C13—C14	−70.1 (2)
C3—O1—C2—C1	61.1 (2)	C15—O2—C14—C13	60.1 (2)
N1—C1—C2—O1	−58.5 (2)	N2—C13—C14—O2	−57.5 (2)
C2—O1—C3—C4	−60.1 (2)	C14—O2—C15—C16	−61.0 (2)
O1—C3—C4—N1	56.6 (2)	C24—N2—C16—C15	−171.17 (16)
C12—N1—C4—C3	71.1 (2)	C13—N2—C16—C15	−53.3 (2)
C1—N1—C4—C3	−51.1 (2)	C17—N2—C16—C15	66.7 (2)
C5—N1—C4—C3	−169.51 (17)	O2—C15—C16—N2	59.0 (2)
C12—N1—C5—C6	−56.4 (2)	C24—N2—C17—C18	−57.3 (2)
C1—N1—C5—C6	66.1 (2)	C16—N2—C17—C18	63.9 (2)
C4—N1—C5—C6	−177.22 (17)	C13—N2—C17—C18	−176.85 (17)
N1—C5—C6—C7	101.2 (2)	N2—C17—C18—C19	−80.6 (2)
N1—C5—C6—C11	−84.4 (2)	N2—C17—C18—C23	105.1 (2)
C11—C6—C7—C8	0.2 (3)	C23—C18—C19—C20	−1.9 (3)
C5—C6—C7—C8	174.8 (2)	C17—C18—C19—C20	−176.3 (2)
C6—C7—C8—C9	0.0 (4)	C18—C19—C20—C21	2.5 (3)
C7—C8—C9—C10	0.2 (4)	C19—C20—C21—C22	−1.1 (3)
C8—C9—C10—C11	−0.6 (4)	C20—C21—C22—C23	−0.9 (3)
C9—C10—C11—C6	0.8 (4)	C21—C22—C23—C18	1.5 (3)
C7—C6—C11—C10	−0.6 (3)	C19—C18—C23—C22	−0.1 (3)
C5—C6—C11—C10	−175.1 (2)	C17—C18—C23—C22	174.43 (19)
